# Epidemic Characteristics and Meteorological Risk Factors of Hemorrhagic Fever With Renal Syndrome in 151 Cities in China From 2015 to 2021: Retrospective Analysis

**DOI:** 10.2196/52221

**Published:** 2024-06-05

**Authors:** Yizhe Luo, Longyao Zhang, Yameng Xu, Qiyuan Kuai, Wenhao Li, Yifan Wu, Licheng Liu, Jiarong Ren, Lingling Zhang, Qiufang Shi, Xiaobo Liu, Weilong Tan

**Affiliations:** 1 Department of Epidemiology School of Public Health Nanjing Medical University Nanjing China; 2 Nanjing Bioengineering (Gene) Technology Center for Medicines Nanjing China; 3 Department of Biostatistics School of Public Health Nanjing Medical University Nanjing China; 4 Jiangsu Macro and Micro Test Med-tech Co, Ltd Nantong China; 5 National Key Laboratory of Intelligent Tracking and Forecasting for Infectious Diseases, National Institute for Communicable Disease Control and Prevention, Chinese Center for Disease Control and Prevention, Beijing, China Beijing China; 6 College of Life Science Fujian Agriculture and Forestry University Fuzhou China; 7 Department of Vector Control School of Public Health Shandong University Jinan China; 8 Xinjiang Key Laboratory of Vector-borne Infectious Diseases Urumqi China

**Keywords:** China, hemorrhagic fever with renal syndrome, HFRS, climate change, meteorological factors, distributed lag nonlinear model

## Abstract

**Background:**

Hemorrhagic fever with renal syndrome (HFRS) continues to pose a significant public health threat to the population in China. Previous epidemiological evidence indicates that HFRS is climate sensitive and influenced by meteorological factors. However, past studies either focused on too-narrow geographical regions or investigated time periods that were too early. There is an urgent need for a comprehensive analysis to interpret the epidemiological patterns of meteorological factors affecting the incidence of HFRS across diverse climate zones.

**Objective:**

In this study, we aimed to describe the overall epidemic characteristics of HFRS and explore the linkage between monthly HFRS cases and meteorological factors at different climate levels in China.

**Methods:**

The reported HFRS cases and meteorological data were collected from 151 cities in China during the period from 2015 to 2021. We conducted a 3-stage analysis, adopting a distributed lag nonlinear model and a generalized additive model to estimate the interactions and marginal effects of meteorological factors on HFRS.

**Results:**

This study included a total of 63,180 cases of HFRS; the epidemic trends showed seasonal fluctuations, with patterns varying across different climate zones. Temperature had the greatest impact on the incidence of HFRS, with the maximum hysteresis effects being at 1 month (–19 ºC; relative risk [RR] 1.64, 95% CI 1.24-2.15) in the midtemperate zone, 0 months (28 ºC; RR 3.15, 95% CI 2.13-4.65) in the warm-temperate zone, and 0 months (4 ºC; RR 1.72, 95% CI 1.31-2.25) in the subtropical zone. Interactions were discovered between the average temperature, relative humidity, and precipitation in different temperature zones. Moreover, the influence of precipitation and relative humidity on the incidence of HFRS had different characteristics under different temperature layers. The hysteresis effect of meteorological factors did not end after an epidemic season, but gradually weakened in the following 1 or 2 seasons.

**Conclusions:**

Weather variability, especially low temperature, plays an important role in epidemics of HFRS in China. A long hysteresis effect indicates the necessity of continuous intervention following an HFRS epidemic. This finding can help public health departments guide the prevention and control of HFRS and develop strategies to cope with the impacts of climate change in specific regions.

## Introduction

Hemorrhagic fever with renal syndrome (HFRS) is a rodent-borne zoonotic disease caused by *Hantavirus* (HTNV), causing symptoms such as fever, headache, and renal dysfunction [1]. Globally, China is one of the countries most affected by HFRS, with cases covering 31 provinces, municipalities, and autonomous regions and numbering nearly 10,000 per year in the past decade [2]. HFRS is listed as a class B infectious disease due to its potential threat to public health in China [3]. Despite the implementation of vaccination programs, HFRS remains a serious public health problem in China. More than 10 HTNV-potential hosts have been identified with population levels sufficient to sustain virus viability and reproduction in nature [4]. Moreover, the enormous geographic differences and the variety of climate types in China make it difficult or impossible to eliminate HFRS, where 9187 new cases were reported in 2021, including 64 deaths [5].

Known risk factors for HFRS incidence include climate, host population, and viral dynamics [6]. Climate is widely recognized as a key factor in HTNV transmission, mainly affecting the prevalence of the virus and the risk of human infection by affecting rodent population dynamics [7,8]. For example, temperature, humidity, and precipitation can affect crop yields, which are a food source for rodents [7]. Earlier studies showed that climate-related HFRS outbreaks had a hysteresis effect, usually delayed by 1 to 6 months, and seasonal patterns of HFRS epidemics also showed sensitivity to climate [9-11]. Using a distributed lag nonlinear model (DLNM), Luo et al [12] investigated temperature (lag=6 months, relative risk [RR] 3.05) and precipitation (lag=0 months, RR 2.08), which had the greatest impact on the incidence of HFRS. Sun et al [7] also identified extremely high or low temperature as being strongly associated with HFRS. Concerns have been raised in recent years about the expansion of HFRS-affected areas and the reemergence of HFRS in regions where it had been eliminated. Under global warming, cyclic dynamics of rodent populations are changing, and new endemic areas are forming [13]. There is an urgent need to explore the propagation of HFRS under different climate conditions. Cao et al [14] explored the interactions and marginal effects of meteorological factors on HFRS in different climate zones in 254 cities in China. However, the time span of their study was 2006 to 2016, which is too far in the past; few or no studies have discussed the relationship between HFRS and meteorological factors in different regions in recent years [15].

Based on HFRS monitoring data from 151 cities from 2015 to 2021, we estimated the hysteresis effects and interactions of meteorological factors in different climate zones on HFRS in China. We also sought to identify associations between temperature and HFRS under different environmental conditions and examine whether these associations varied geographically. Compared with previous studies, our research covers a wider range of subjects, is more recent, and has more convincing results.

## Methods

### Ethical Considerations

The study was approved by the ethical review board of the Nanjing Bioengineering (Gene) Technology Center for Medicines (2022005). Consent to participate was not applicable because this study used HFRS surveillance data. All participant data were anonymized and kept confidential to protect the privacy of participants.

### Study Sites

This study was based on a national database of meteorological factors and confirmed HFRS case counts in 151 Chinese prefecture-level cities from January 1, 2015, to December 31, 2021. According to China’s national reporting system for infectious diseases, the total number of HFRS cases in these cities during the period they were included in the study exceeded 50.

China can be divided into 6 climate zones (Multimedia Appendix 1, Figure S1) [16]. In this study, 3 climatic zones were chosen as the research zones: the midtemperate zone, the warm-temperate zone, and the subtropical zone. The cold-temperate zone, the plateau-temperate zone, and the tropical zone were excluded due to having too few cities (<5 cities). A final total of 151 Chinese prefecture-level cities were included in our study (Multimedia Appendix 1, Table S1). When the city boundary spanned multiple climatic zones, the urban climatic zones were divided according to the location of the city center.

### Collection of Data

Monthly data on HFRS from the 151 prefecture-level cities across China from January 1, 2015, to December 31, 2021, including the number of cases and incidence, were provided by the Chinese Center for Disease Control and Prevention. All notified HFRS cases were confirmed according to the united diagnostic criteria issued by the Ministry of Health of China in 1998 [17].

Monthly meteorological data including average temperature (Celsius), average relative humidity (%), and average precipitation (mm) in the selected cities were obtained from 839 meteorological monitoring stations [18] (Multimedia Appendix 1, Figure S2).

### Statistical Analysis

In the descriptive analysis, the mean (SD), median (IQR), and range were used to describe the distribution of cases of HFRS and weather variables in the 3 selected climatic zones. In 2018, there were some missing values for meteorological variables in 18 cities, including mean temperature (n=216), relative humidity (n=216) and precipitation (n=216). Thus, we imputed the values of each city using their last year’s value. The descriptive statistics before and after imputing are summarized in Table 1.

**Table 1:** Descriptive analysis of monthly mean temperature, precipitation, and average relative humidity in different climate zones in China from 2015 to 2021. There were 12 missing values in the midtemperate zone, 60 in the warm-temperate zone, and 144 in the subtropical zone.

Zones and factors				Values		Imputed values
**Midtemperate zone (n=25 cities)**						
	**HFRS^a^ cases (n)**					
		Mean (SD)	6 (7)		—^b^	
		Median (IQR)	4 (2 to 8)		—	
		Range	0 to 79		—	
	**Temperature (ºC)**					
		Mean (SD)	5.43 (13.67)		5.42 (13.68)	
		Median (IQR)	7.49 (–6.93 to 17.36)		7.47 (6.96 to 17.36)	
		Range	–31.14 to 26.94		–31.14 to 26.94	
	**Precipitation (mm)**					
		Mean (SD)	55.28 (67.41)		55.22 (67.32)	
		Median (IQR)	27.70 (8.90 to 79.55)		27.70 (8.90 to 79.55)	
		Range	0.00 to 677.20		0.00 to 677.20	
	**Relative humidity (%)**					
		Mean (SD)	64.53 (11.84)		64.55 (11.82)	
		Median (IQR)	65 (56.86 to 73)		25 (56.93 to 65.02)	
		Range	25 to 93		25 to 93	
**Warm-temperate zone (n=54 cities)**						
	**HFRS cases (n)**					
		Mean (SD)	6 (22)		—	
		Median (IQR)	2 (0 to 5)		—	
		Range	0 to 878		—	
	**Temperature (ºC)**					
		Mean (SD)	13.44 (10.08)		13.43 (10.08)	
		Median (IQR)	14.25 (4.33 to 22.59)		14.25 (4.32 to 22.59)	
		Range	–10.96 to 30.48		–10.96 to 30.48	
	**Precipitation (mm)**					
		Mean (SD)	63.14 (127.24)		63.65 (136.24)	
		Median (IQR)	33.00 (9.50 to 78.30)		32.90 (9.30 to 3450.80)	
		Range	0.00 to 3450.80		32.90 (9.30 to 3450.80)	
	**Relative humidity (%)**					
		Mean (SD)	63.63 (12.38)		63.52 (12.42)	
		Median (IQR)	63.87 (55 to 73)		63.87 (54.69 to 73)	
		Range	23.32 to 94.42		23.32 to 94.42	
**Subtropical zone (n=72 cities)**						
	**HFRS cases (n)**					
		Mean (SD)	4 (6)		—	
		Median (IQR)	2 (1 to 5)		—	
		Range	0 to 100		—	
	**Temperature (ºC)**					
		Mean (SD)	18.68 (7.94)		18.70 (7.94)	
		Median (IQR)	19.17 (12.33 to 25.85)		19.19 (12.35 to 25.84)	
		Range	–0.78 to 31.95		–0.78 to 31.95	
	**Precipitation (mm)**					
		Mean (SD)	128.87 (123.11)		128.59 (122.98)	
		Median (IQR)	95.35 (45.27 to 178.53)		95.15 (45.00 to 178.12)	
		Range	0.00 to 3450.40		0.00 to 3450.40	
	**Relative humidity (%)**					
		Mean (SD)	77.03 (7.57)		76.96 (7.61)	
		Median (IQR)	77.79 (72.5 to 82.10)		77.68 (72.36 to 82)	
		Range	31 to 97		31 to 97	

^a^HFRS: hemorrhagic fever with renal syndrome.

^b^Not applicable.

### First Stage Analysis

We first captured the association between weather conditions and HFRS incidence in different climate zones with a DLNM, which can flexibly describe relationships and explore underlying lag nonlinear effects [19]. The climate-specific model with adjustment for potential confounders used equation (1):

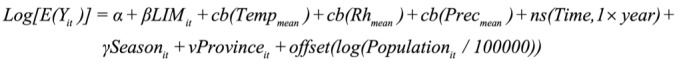



*E (Y_it_)* denotes the monthly expected number of cases in the city and month *t*, and α is the intercept. *LIM_it_* represents the previous month’s incidence, used to reduce autocorrelation between values. *cb(Temp_mean_)*, *cb(Rh_mean_)*, and *cb(Prec_mean_)* are cross-basis matrices of selected monthly meteorological factors, constructed using B-splines for exposure and natural cubic splines for lag (maximum lag of 6 month) dimensions, respectively. A natural cubic spline function of 1 degree of freedom per year was used to control the long-term trend of incidence. The *Time* variable indicates the sequence (from 1 to 84) with month as the unit during the study period, 2015 to 2021. *Season_it_* and *Province_it_* and are categorical variables to control seasonal patterns and different provinces, respectively. The offset term is the population of each city.

### Second Stage Analysis

In the second stage, the correlation of HFRS and climate under different hysteresis conditions is examined. Exposure-effect curves for climate variables with different lag times in the 3 selected temperature zones were drawn to illustrate the hysteresis effects and their duration under different meteorological conditions. Furthermore, the HFRS-climate association at temperature extremes was explored. Taking the median of different meteorological conditions as the reference, the relationship between the corrected meteorological conditions at the 2.5th and 97.5th percentiles and the incidence of HFRS were calculated, respectively. Finally, a random-effect meta-analysis of city-specific relationships for different climate zones estimated in the first stage was performed using the restricted maximum likelihood estimation method to provide more accurate estimates.

### Third Stage Analysis

To explore the interaction and stratification effects between the 3 weather condition and HFRS epidemics in different climate zones, we constructed a generalized additive model (GAM) in the third stage. The model can be written as equation (2):

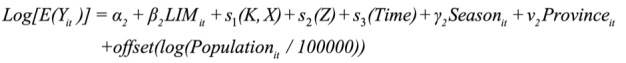



Here, α_2_ is the intercept; *K* denotes 1 of the weather conditions (mean temperature, relative humidity, and precipitation), and *X* and *Z* denote the other 2 indicated penalized spline functions. *s_1_(K,X)* is the spline function of the interaction between variables *K* and *X*.

Then, the meteorological stratification between mean temperature and the incidence of HFRS was determined for relative humidity and precipitation. We split relative humidity and precipitation into category variables, including the medians for “high” and “low.”

### Sensitivity Analysis

To test whether our main conclusions were robust, we performed a sensitivity analysis relying on the quasi-Akaike information criterion (QAIC) and quasi-Bayesian information criterion (QBIC). We used QAIC and QBIC to identify the optimal number and location of knots for the natural spline and the optimal number of lag months from 1 to 6 (Multimedia Appendix 1, Table S2).

R (version 4.2.1; R Foundation for Statistical Computing) and the R packages *dlnm*, *metafor*, and *mcgv* were used for constructing the DLNM and GAM based on the 3 meteorological variables. All maps were created using ArcGIS (version 10.2; Esri Inc). The confidence level of all 2-sided statistical tests in this study was set at 95%, and the significance level was set at .05.

## Results

### Descriptive Analysis

**Figure 1:**
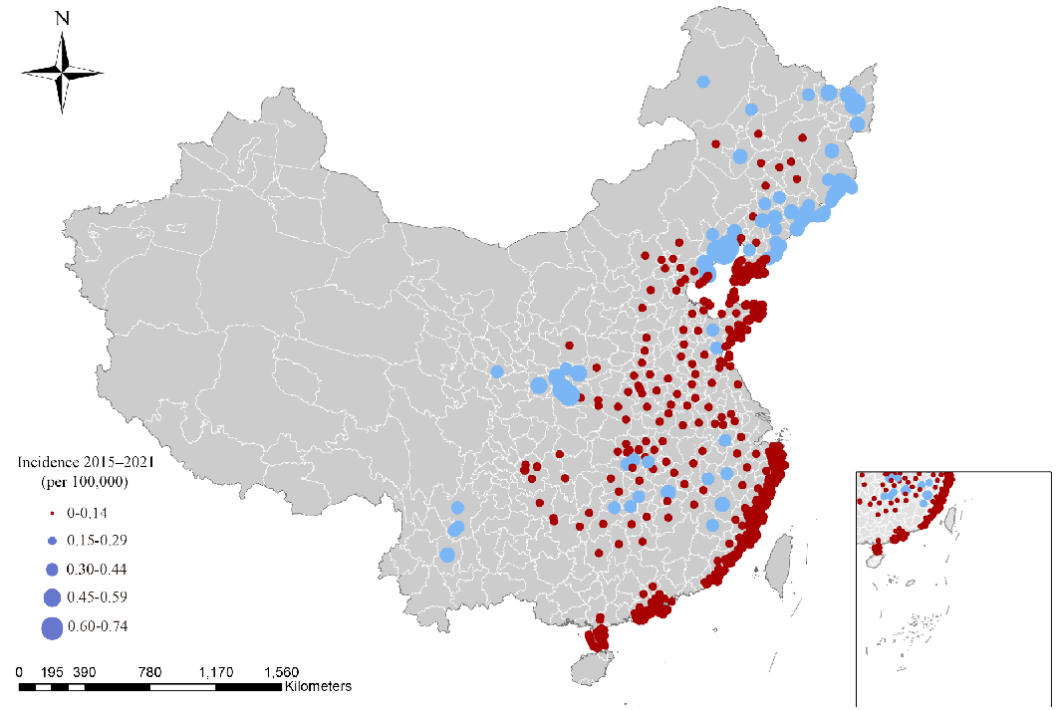
Distribution of cases of hemorrhagic fever with renal syndrome in China from 2015 to 2021.

A total of 63,180 HFRS cases were involved in our study (Figure 1). Between 2015 and 2021, 12,481, 28,353, and 22,346 cases of HFRS occurred in the midtemperate zone, warm-temperate zone, and subtropical zone. Multimedia Appendix 1, Figure S3 shows bar charts of different seasonal prevalence patterns of HFRS in the 3 climate zones. The peaks for HFRS cases in the midtemperate, warm-temperate, and subtropical zones occurred in autumn; winter and spring; and winter and spring, respectively. The mean values for temperature, precipitation, and relative humidity in the 3 zones were 5.43 °C, 55.28 mm, and 64.53%; 13.44 °C, 63.14 mm, and 63.63%; and 18.68 °C, 128.8 mm, and 77.03%, respectively. Thus, there was a gradual increase from north to south.

### DLNM Analysis

**Figure 2:**
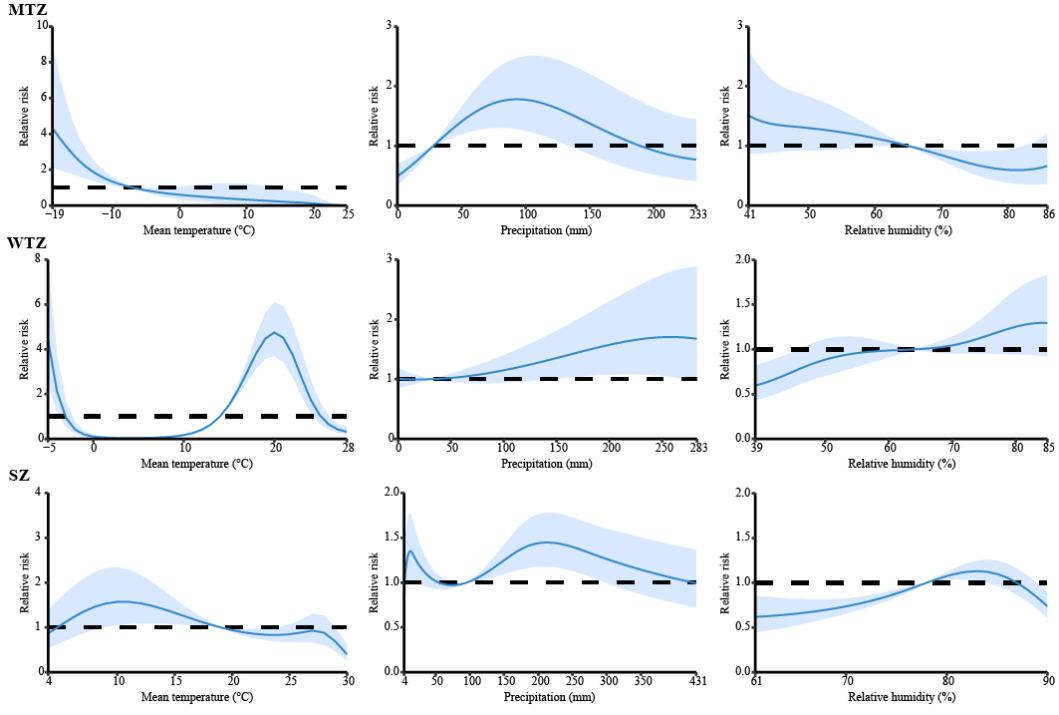
Summary of cumulative exposure-response curves of hemorrhagic fever with renal syndrome incidence for meteorological factors with a lag of 0-6 months in 3 selected temperature zones from 2015 to 2021. The y-axis represents the relative risk of each variable. The x-axis represents the range of observations for each variable. The blue lines represent means estimated by the distributed lag nonlinear model, and the shaded areas represent the 95% CI. MTZ: midtemperate zone; SZ: subtropical zone; WTZ: warm temperate zone.

**Figure 3:**
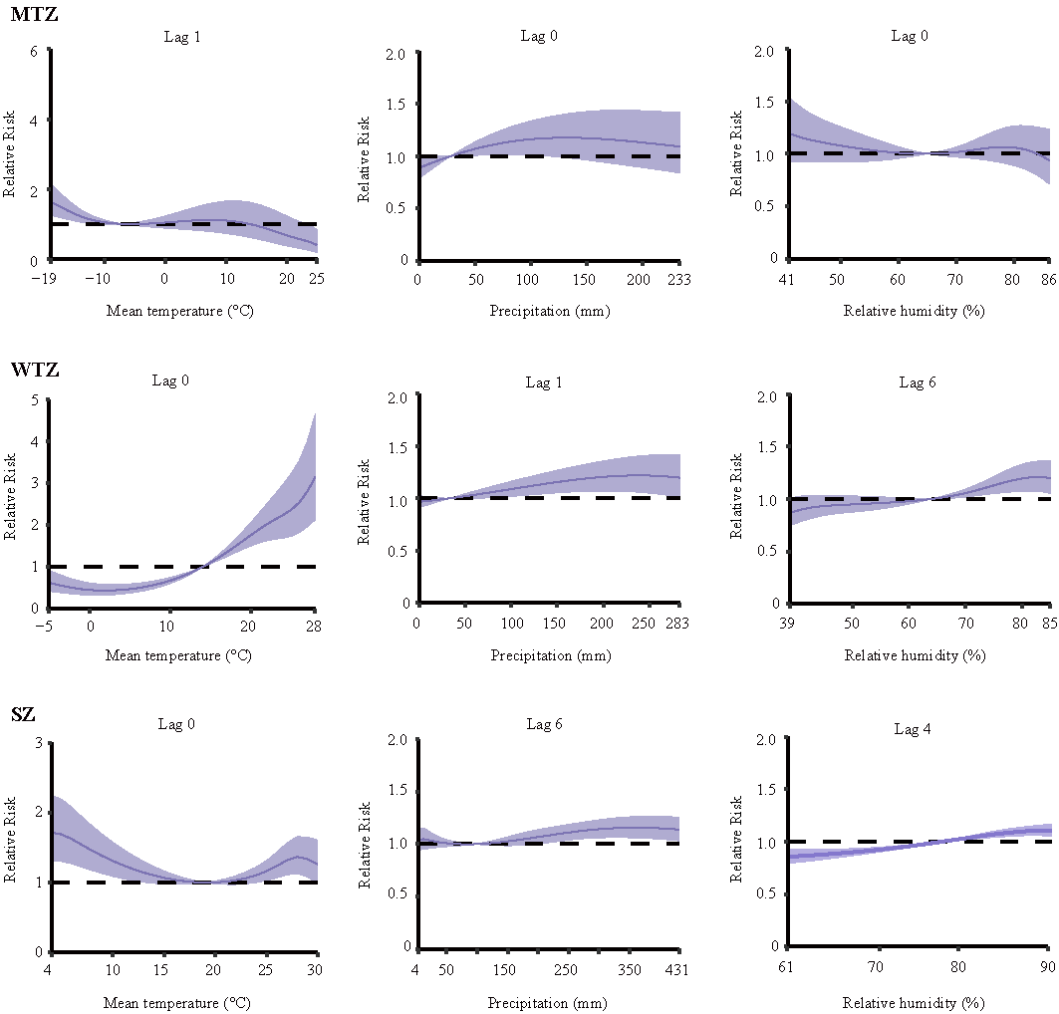
Lag-specific effects of meteorological factors on hemorrhagic fever with renal syndrome infection in different climate zones from 2015 to 2021. The y-axis represents the relative risk of each variable. The x-axis represents the range of observations for each variable. The purple lines represent means estimated by the distributed lag nonlinear model, and the shaded areas represent the 95% CI. MTZ: midtemperate zone; SZ: subtropical zone; WTZ: warm temperate zone.

**Figure 4:**
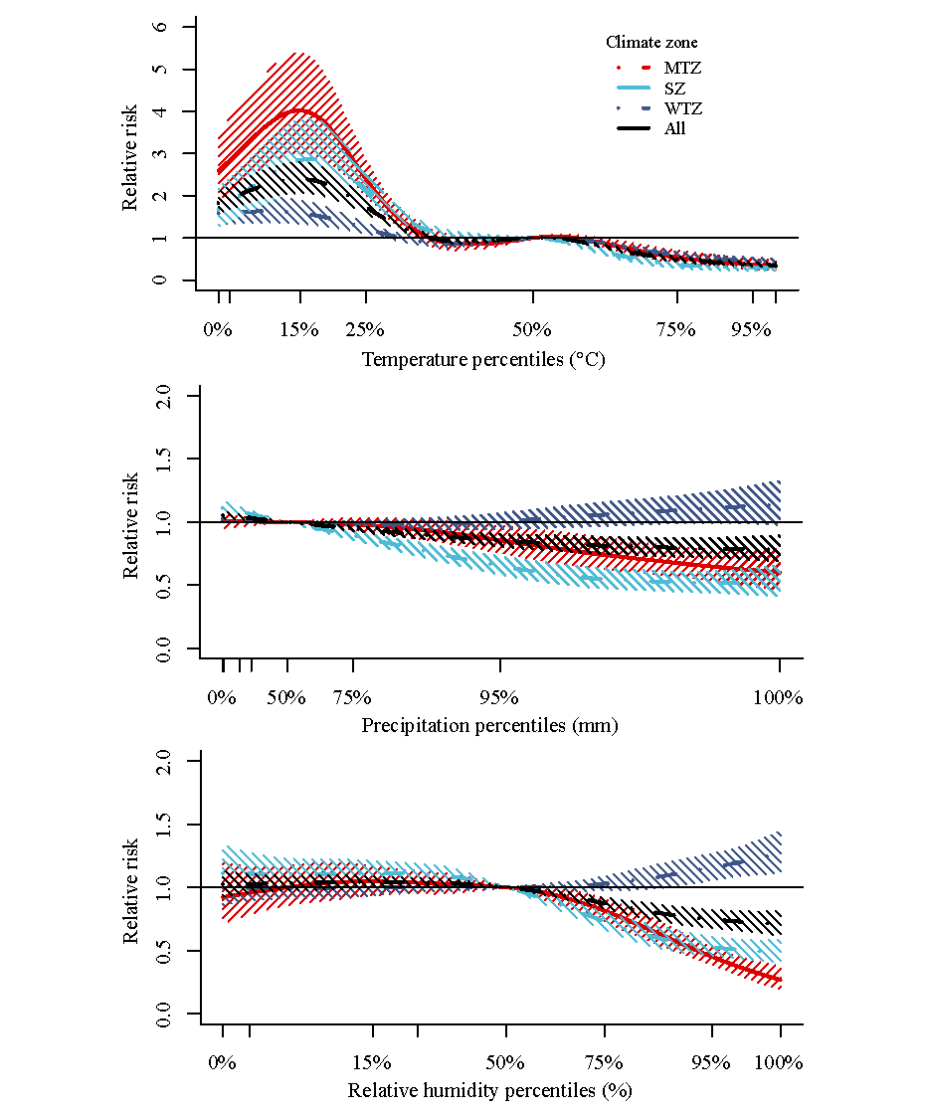
Meta-analysis of meteorological factors on hemorrhagic fever with renal syndrome in different climate zones from 2015 to 2021. “All” represents data from all 151 cities, covering all temperature zones. MTZ: midtemperate zone; SZ: subtropical zone; WTZ: warm temperate zone.

The cumulative risk between meteorological factors and HFRS incidence in the DLNM for different climate zones in China after controlling for seasonal and long-term trends is shown in Figure 2. In the midtemperate zone, the meteorological conditions that were positively correlated with HFRS risk were mean temperature <–7 ° and precipitation 28 to 134 mm; in the warm-temperate zone, the meteorological conditions were a mean temperature <–7 °C or 14 °C to 24 °C and precipitation 143 mm to 274 mm; in the subtropical zone, the meteorological conditions were mean temperature 9 °C to 19 °C, precipitation 11 mm to 22 mm or 95 mm to 299 mm, and relative humidity 78% to 84%. Figure 3 and Multimedia Appendix 1, Figure S4 show the impact of different lag months on climate-related HFRS risk. In the midtemperate zone, significant RRs were observed at lag of 1 month when mean temperature was –19 °C (RR 1.64, 95% CI 1.24-2.15). In the warm-temperate zone, a temperature of 28 °C (0-month lag; RR 3.15, 95% CI 2.13-4.65), precipitation of 239 mm (1-month lag; RR 1.22, 95% CI 1.06-1.40), and relative humidity of 83% (6-month lag; RR 1.21, 95% CI 1.07-1.36) resulted in significantly higher RR. In addition, in the subtropical zone, temperature, precipitation, and relative humidity with lags of 0, 6, and 4 months had high RRs at 4°C (RR 1.72, 95% CI 1.31-2.25), 360 mm (RR 1.16, 95% CI 1.06-1.26), and 90% (RR 1.11, 95% CI 1.05-1.17), respectively. Multimedia Appendix 1, Figure S5 shows the RR between climate and HFRS with different lag months for extreme weather. In the midtemperate zone and subtropical zone, HFRS was sensitive to low temperature, while in the warm-temperate zone, HFRS was more sensitive to high temperature. Higher precipitation and humidity were associated with the incidence of HFRS in the warm temperate zone and subtropical zone. Moreover, the results of the meta-analysis showed that low temperature, relatively high precipitation, and high relative humidity were risk factors for the onset of HFRS, but the subtropical zone showed a different trend (Figure 4).

### Interaction and Stratified Analysis

The results for 25 cities in the midtemperate zone showed that in environments with low temperature and low relative humidity and in environments with low temperature and high relative humidity, each decrease of 1 °C led to an increase in the risk of HFRS of 3% (95% CI 2.6%-3.4%) and 4.6% (95% CI 3.8%-5.4%), respectively. In environments with low temperature and low precipitation and in environments with low temperature and high precipitation, each 1 °C decrease led to an increase in the risk of HFRS of 3.3% (95% CI 2.8%-3.9%) and 5.3% (95% CI 4.4%-6.1%), respectively. The results from 54 cities in the warm-temperate zone showed that in environments with low temperature and low relative humidity and in environments with low temperature and high relative humidity, each decrease of 1 °C led to an increase in the risk of HFRS of 1.8% (95% CI 1.5%-2.2%) and 4.7% (95% CI 3.8%-5.7%), respectively. In environments with low temperature and low precipitation and in environments with low temperature and high precipitation, each decrease of 1 °C led to an increase in the risk of HFRS of 2.3% (95% CI 2%-2.7%) and 2.4% (95% CI 1.6%-3.3%), respectively (Table 2 and Multimedia Appendix 1, Table S3-S12).

**Table 2:** Effect of each increase or decrease of 1 °C on hemorrhagic fever with renal syndrome at different temperature levels in different climate zones in China from 2015 to 2021. The low, middle, and high temperature levels in the 3 climate zones were set based on the 2.5th, 50th, and 97.5th percentiles, respectively.

Temperature zones		Relative humidity, relative risk (95% CI)				Precipitation, relative risk (95% CI)	
		Low		High		Low	High
**Midtemperate zone**							
	Low temperature (–19 ºC)^a^		1.030 (1.034-1.026)		1.046 (1.054-1.038)	1.033 (1.039-1.028)	1.053 (1.061-1.044)
	Middle temperature (7 ºC)		0.971 (0.976-0.965)		0.956 (0.952-0.961)	0.968 (0.974-0.962)	0.950 (0.945-0.955)
	High temperature (25 ºC)		0.971 (0.978-0.963)		0.956 (0.958-0.955)	0.968 (0.976-0.960)	0.950 (0.952-0.949)
**Warm-temperate zone**							
	Low temperature (–5 ºC)^a^		1.018 (1.022-1.015)		1.047 (1.057-1.038)	1.023 (1.027-1.020)	1.024 (1.033-1.016)
	Middle temperature (14 ºC)		0.982 (0.986-0.978)		0.955 (0.951-0.959)	0.977 (0.982-0.972)	0.977 (0.975-0.978)
	High temperature (28 ºC)		0.982 (0.990-0.975)		0.955 (0.956-0.953)	0.977 (0.985-0.969)	0.977 (0.981-0.972)
**Subtropical zone**							
	Low temperature (4 ºC)^a^		0.969 (0.967-0.971)		0.973 (0.965-0.980)	0.967 (0.966-0.968)	0.974 (0.967-0.981)
	Middle temperature (19 ºC)		0.969 (0.974-0.964)		0.973 (0.970-0.975)	0.967 (0.973-0.962)	0.974 (0.972-0.976)
	High temperature (30 ºC)		0.969 (0.977-0.961)		0.973 (0.976-0.969)	0.967 (0.975-0.960)	0.974 (0.978-0.970)

^a^These values represent the effect of a decrease of 1 ºC, while unmarked values represent the effect of an increase of 1 ºC.

## Discussion

### Principal Findings

In this study, we quantified the HFRS-weather association in 151 selected cities using a DLNM. Previous studies have investigated the HFRS-weather associations at single- or multiple-location levels [14,15]. However, few of them explored the interaction and hysteresis effects of meteorological factors at different levels on the incidence of HFRS in different climate zones. To the best of our knowledge, this is the first study using national data to explore the interaction and hysteresis effects of meteorological factors at different levels on HFRS in China. Our pooled analysis results showed that meteorological factors, especially temperature, had a significant impact on the incidence of HFRS in China, although the association between HFRS and different meteorological factors, the lag time with the greatest impact, and the duration of the lagged effect varied by location. Moreover, we found low temperature was a contributing factor to the pathogenesis of HFRS, and HFRS was relatively more sensitive to temperature changes in the warm- and midtemperate zones than in the subtropical zone. This finding can help public health departments guide the prevention and control of HFRS and develop strategies to cope with the impacts of climate change in specific regions.

We found that low temperature was the most important meteorological factor for the onset of HFRS. It is worth noting that the relationship between HFRS and weather varies by location. There were different impact patterns in the 3 climatic zones. The midtemperate zone peaked at low temperatures (<–7 ºC); the warm-temperate zone peaked at both low temperatures (<–4 ºC) and high temperatures (14-24 ºC); the subtropical zone peaked at 9-19 ºC. The differences may be related to the different host animal species, reproduction, and activity cycles. *Apodemus agrarius* and *Rattus norvegicus* are the main species involved in HFRS transmission in China. HFRS infection caused by *A agrarius* mainly occurs in autumn and winter, while infection caused by *R norvegicus* occurs in spring [14]. Combined with the seasonal patterns, it can be inferred that *A agrarius* is the main vector in the midtemperate zone, while *A agrarius* and *R norvegicus* are the main vectors in the warm-temperate and subtropical zones. The main hosts of HTNV and Seoul virus (SEOV) are *A agrarius* and *R norvegicus*, respectively. It can be inferred that HTNV is epidemic in the midtemperate zone, while HTNV and SEOV are mainly epidemic in the warm-temperate and subtropical zones. Prior studies investigated sites and identified HTNV in the midtemperate Heilongjiang Province, the warm-temperate Qingdao City, and the subtropical Jiangxi Province, which is consistent with our inferences [20-22]. Moreover, low temperatures prolong the survival of the virus outside the host, allowing the virus to remain infectious even in the absence of direct rodent contact or rodent-to-human contact [23].

The results of the DLNM showed that the risk of climate-related HFRS varied from place to place with different lag months. The lagged effects of climate variables in different temperature zones may be related to the dominant rodent population, breeding and living conditions, HTNV-positive rate, and human contact frequency [24,25]. We found that the maximum lag effects of temperature on HFRS incidence were 1 month, 0 months, and 0 months from northern to southern China. However, inconsistent findings on the lag time with maximum effects have also been reported by Cao et al [14]. The lag effects of temperature were 1 month (midtemperate zone), 2 months (warm-temperate zone), and 3 months (subtropical zone), respectively. Our results showed that the hysteresis effect of meteorological factors did not end after one epidemic season, but gradually weakened in the following 1 to 2 epidemic seasons, and the duration of the hysteresis effect varied by region, indicating the necessity of continuous intervention after the HFRS epidemic. At the same time, it also provides theoretical support for the important role of weather variability, especially temperature, in the propagation of HFRS.

Consistent with previous studies, we found that high precipitation and relative humidity are risk conditions for HFRS (Multimedia Appendix 1, Figure S6) [15,26]. Wet conditions and high relative humidity are good for rodents to survive or breed. Adequate rainfall provides a suitable environment and sufficient food for rodents, which ultimately increases the risk of virus transmission [27]. Higher relative humidity affects the spread of HFRS by affecting the infectivity and stability of HTNV in vitro, which is consistent with the fact that HFRS epidemic areas are mostly located in humid or semihumid mountainous areas [28,29]. The interaction and stratification analysis showed that in low-temperature environments, more precipitation and higher relative humidity were climate risk factors for HFRS occurrence, which is consistent with previous studies. Zhang et al [9] found that average temperature, relative humidity, and precipitation interacted with HFRS through stratified analysis; the risk of HFRS was inversely proportional to average temperature and directly proportional to relative humidity.

There are several key limitations to our study that should be acknowledged. First, in addition to meteorological factors, other factors may also affect the occurrence of HFRS, including vaccination programs, economic factors, health care level, and host animal diversity. Second, HFRS case data comes from the notifiable infectious disease detection system, and there are cases of underreporting. For example, patients with mild symptoms may self-isolate at home, which would lead us to underestimate the impact of meteorological factors on HFRS. Third, our data cannot distinguish which viruses caused the HFRS cases, nor can it be targeted to study the relationship between different viruses and climate. Therefore, future research should explore the relationship between HFRS incidence and the different viruses that cause HFRS and explore the intersection between the COVID-19 pandemic and HFRS to fully understand the broader implications for public health.

### Conclusions

Using data for HFRS cases from 151 cities, we provide first-hand evidence of the interaction and stratification effects of meteorological factors on HFRS in different regions of China. Furthermore, the magnitude and timing of hysteresis effects varied across climate zones. Our findings indicate that low temperature positively influences the long-term incidence of HFRS. The results of this study can provide a valuable scientific basis for public health departments to formulate targeted HFRS interventions, understand the relationship between weather and HFRS, and use low temperature as an early warning signal to carry out HFRS control and outbreak response.

## Appendix

Multimedia Appendix 1 Supplementary tables and figures.

## Abbreviations

DLNM: distributed lag nonlinear model
HFRS: hemorrhagic fever with renal syndrome
HTNV: Hantavirus
QAIC: quasi-Akaike information criterion
QBIC: quasi-Bayesian information criterion
RR: relative risk
SEOV: Seoul virus

## Data Availability

The meteorological data are available from the National Meteorological Information Center [18] and HFRS case data are available from the Chinese Center for Disease Control and Prevention [30]. The data set analyzed in this study is available from the corresponding author on reasonable request.
