# Supplementary material for: Epidemic Characteristics and Meteorological Risk Factors of Hemorrhagic Fever With Renal Syndrome in 151 Cities in China From 2015 to 2021: Retrospective Analysis
Source: JMIR Public Health Surveill. 2024 Jun 5;10:e52221. doi: 10.2196/52221 (PMC11187512; doi:10.2196/52221)
Supplement: Multimedia Appendix 1 [file publichealth_v10i1e52221_app1.doc]

**Epidemic Characteristic and Risk Meteorological Factors of Hemorrhagic Fever with Renal Syndrome (HFRS) in 151 cities of China From 2015 to 2021: Retrospective Analysis**
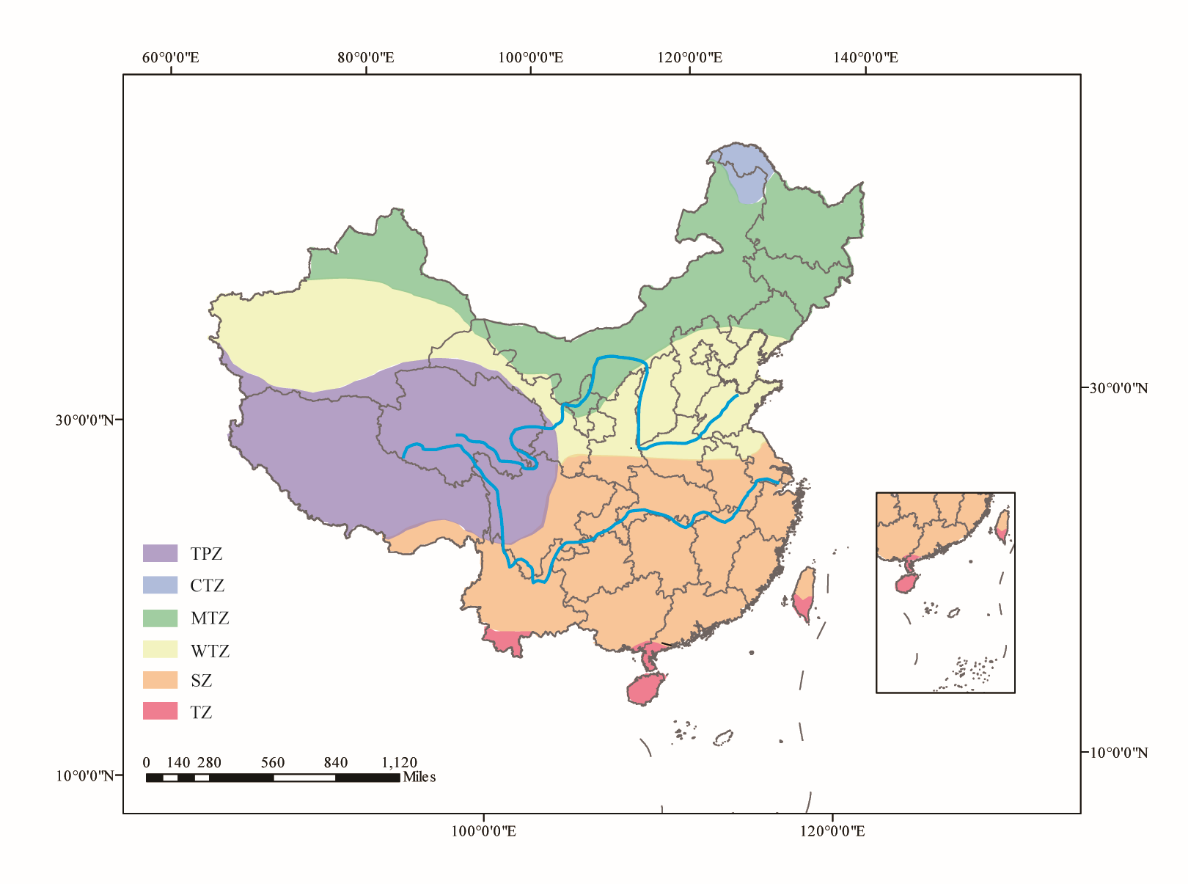


**Fig. S1 Division of climate zones in China**

Notes: TZ = Tropical Zone; SZ = Subtropical Zone; WTZ = Warm Temperate Zone; MTZ = Mid Temperate Zone: CTZ = Cold Temperate Zone: TPZ = Tibetan Plateau Zone


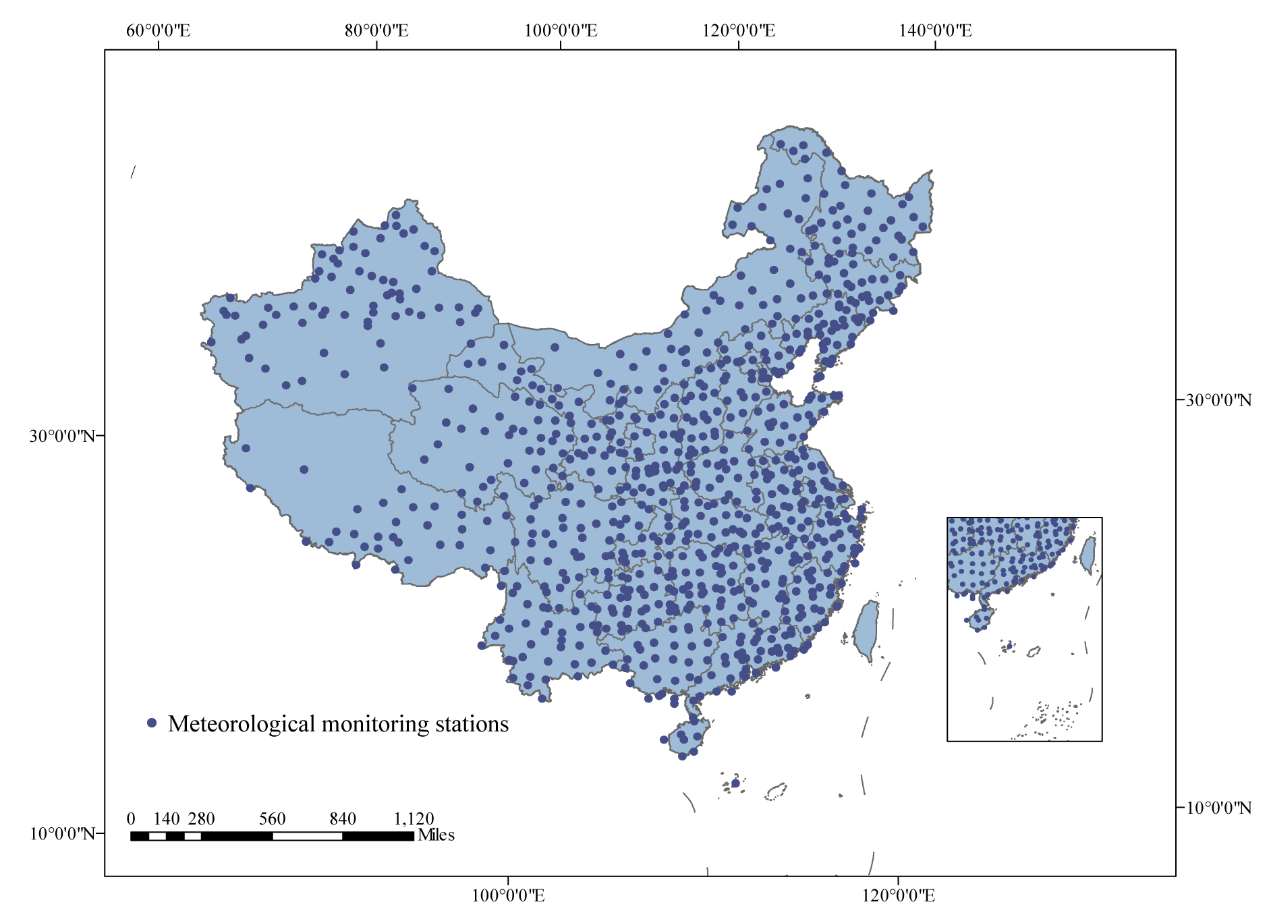


**Fig. S2 Geographical location of meteorological monitoring stations (2015-2021) in China**

Meteorological monitoring stations (N=839). The map was created using ArcGIS 10.2 (Esri Inc, Redlands, CA, USA) (http://desktop.arcgis.com).

**
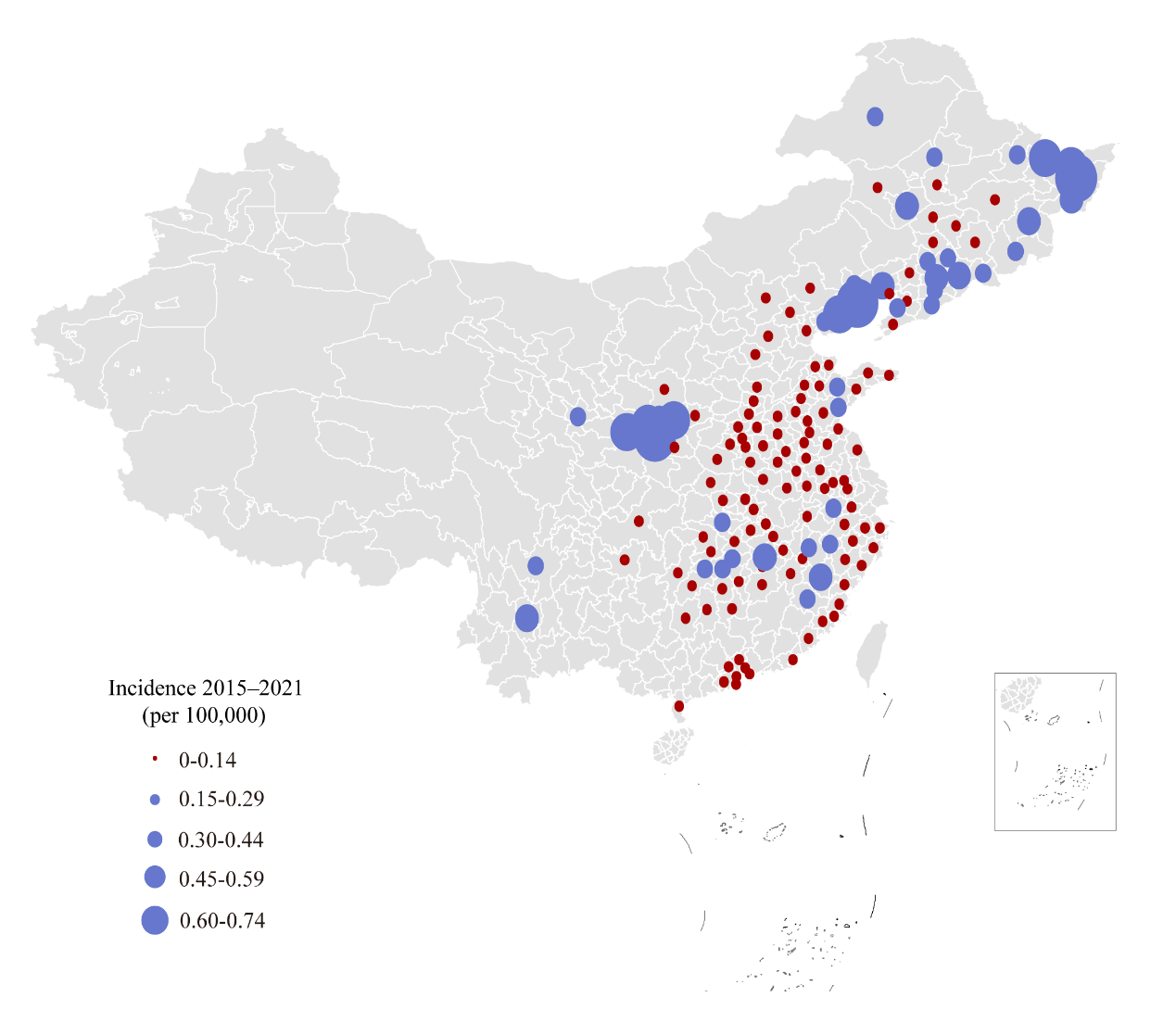
**

**Fig. S3 Distribution of HFRS cases in China, 2015-2021**


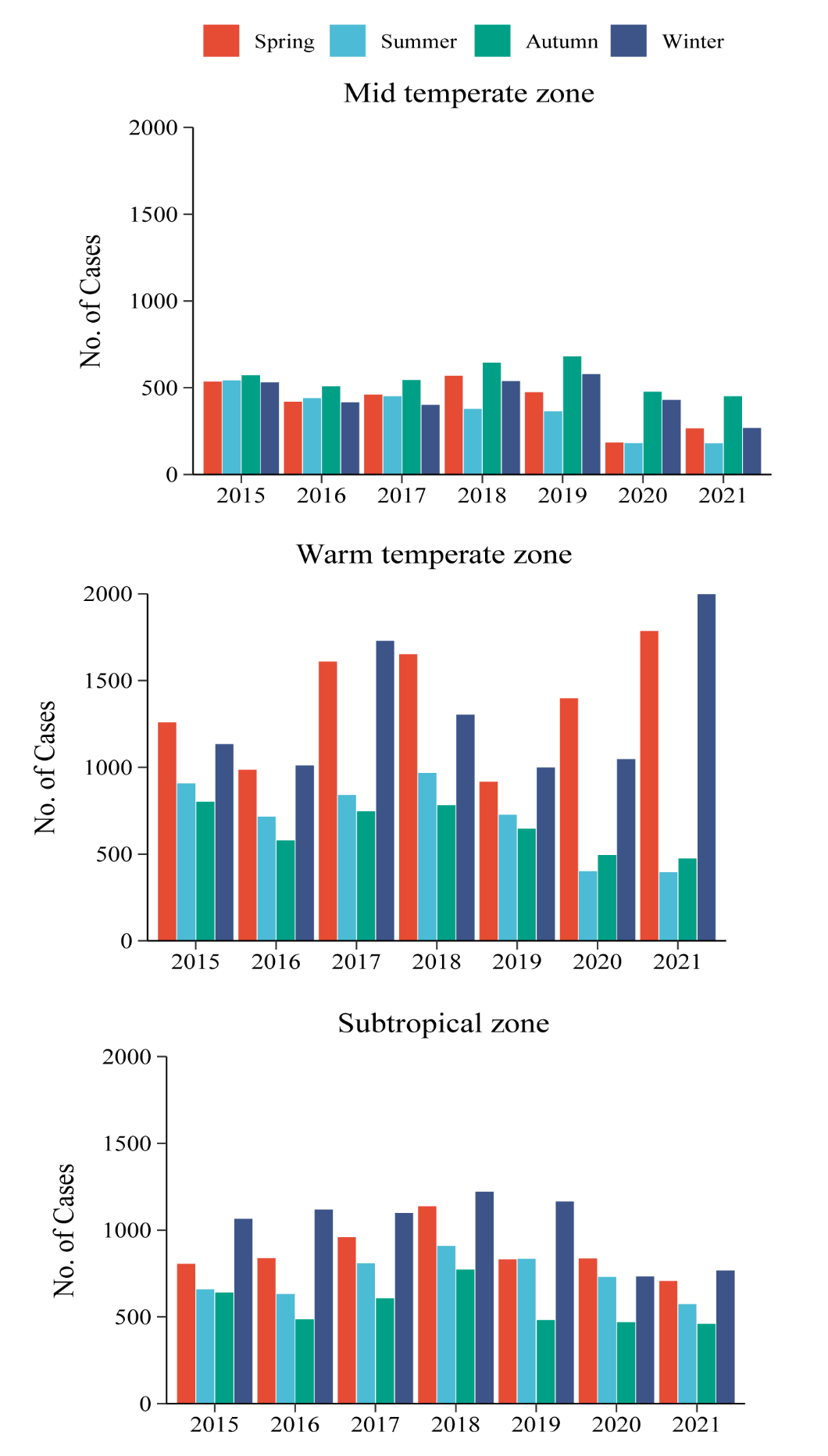


**Fig. S4 Temporal variation of the monthly number of HFRS cases in different climate zones, 2015–2021**


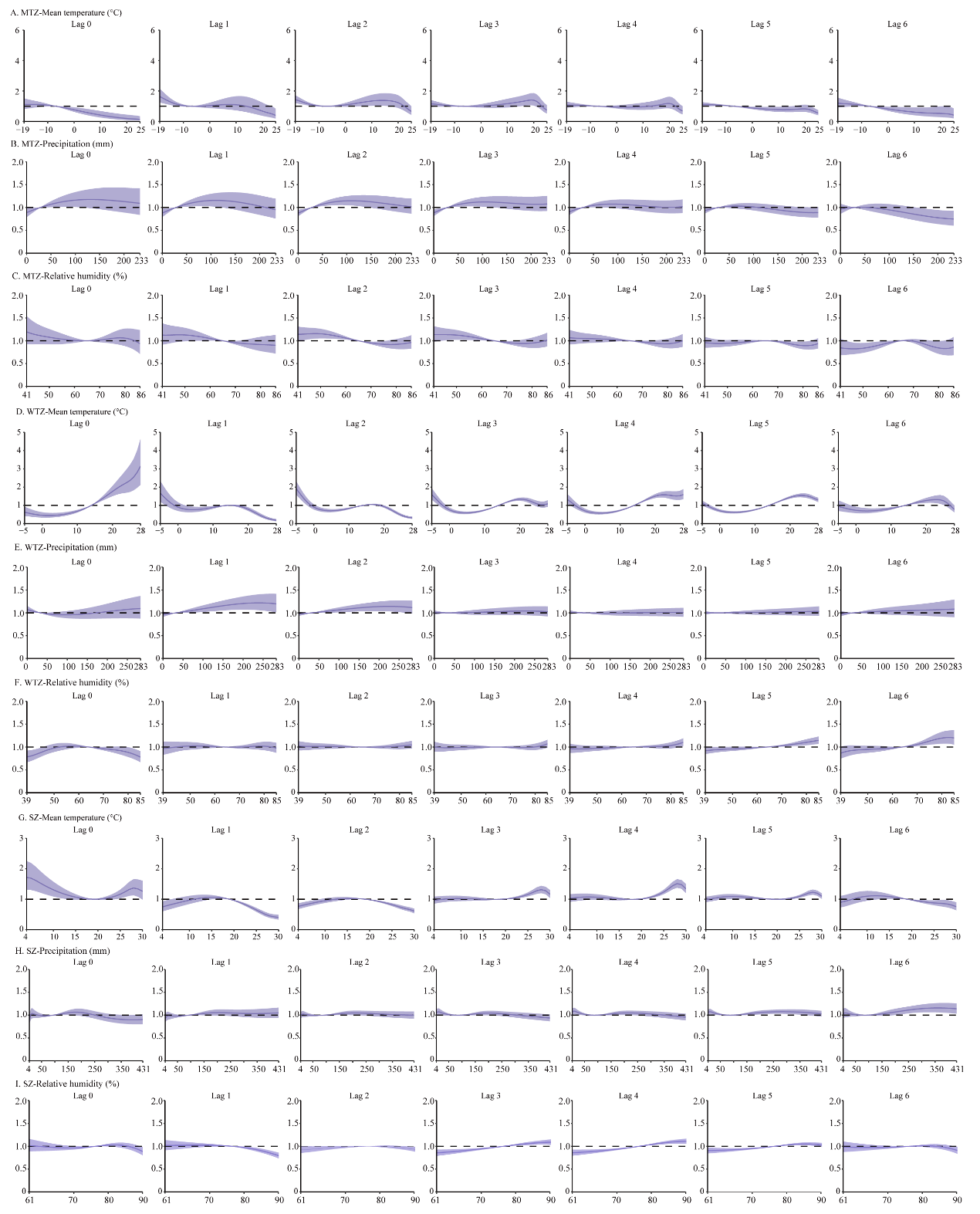


**Fig. S5 Lag-specific effects of meteorological factors on HFRS infection in different climate zones, 2015–2021.**


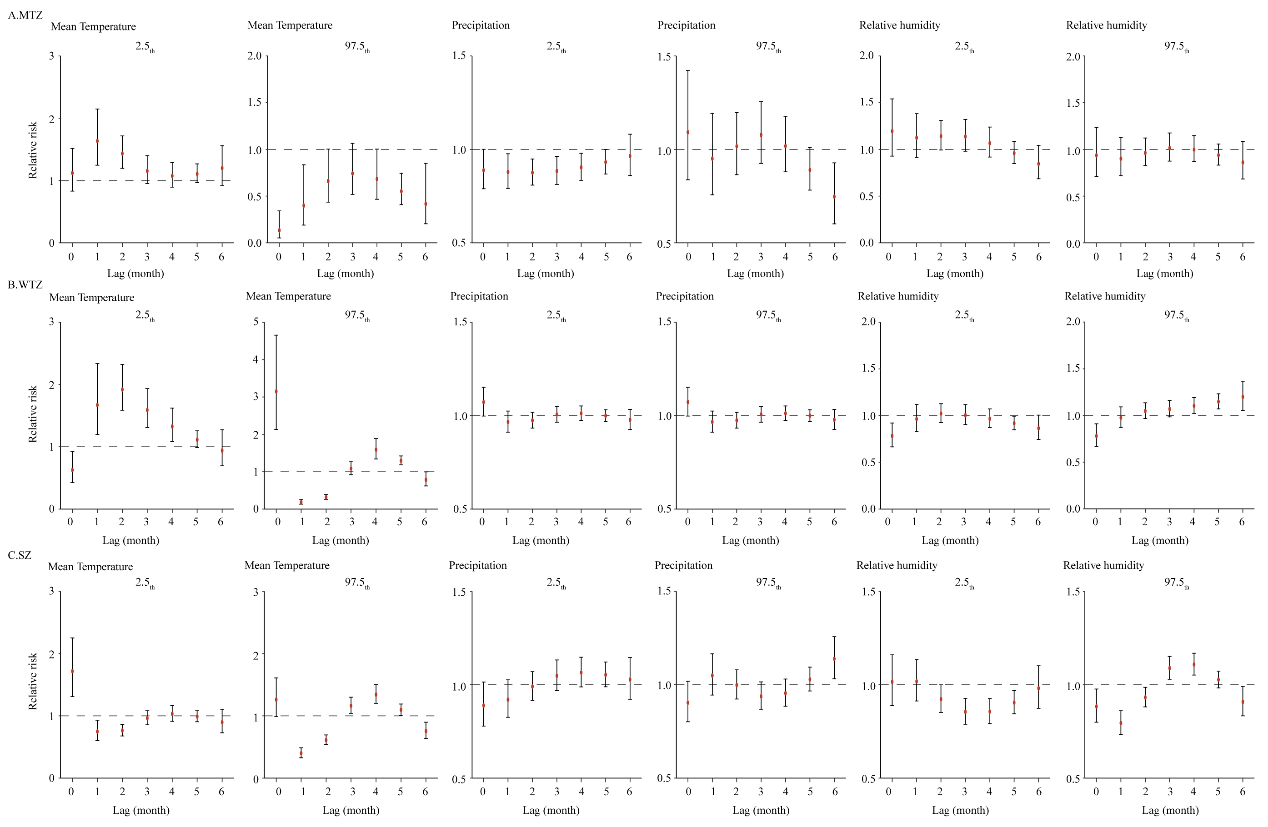


**Fig. S6 The lag effect between mean temperature, precipitation, relative humidity and HFRS infection.**

The error bars mean confidence interval.


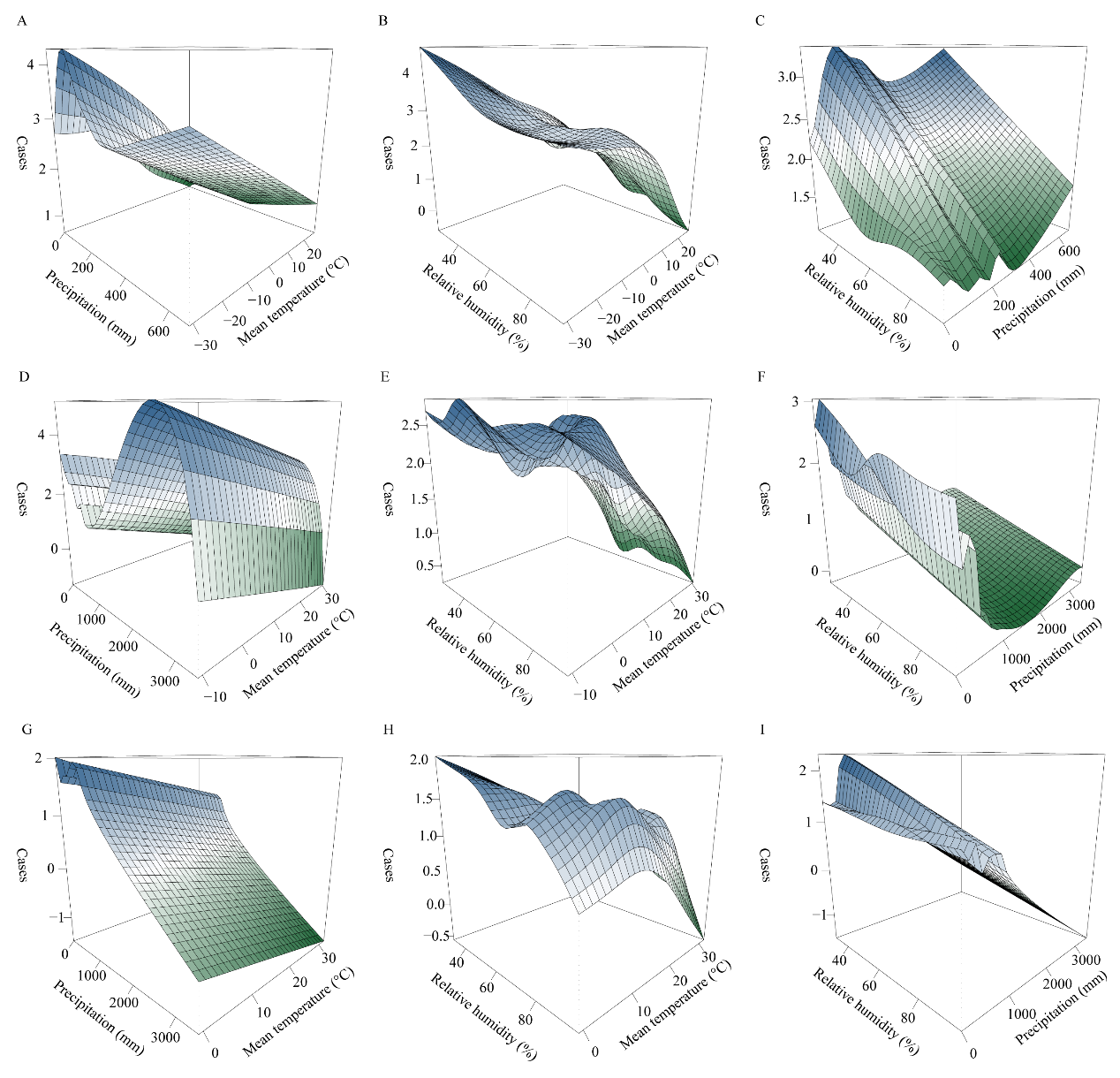


**Fig. S7:** **The effect interactions of the association among meteorological factors and HFRS infection**

**Table S1: The included 151 cities in the analysis**

| **Province** | **City** | **Site number** |
| --- | --- | --- |
| Guangdong | Guangzhou | 59287 |
|  | Foshan | 59298 |
|  | Zhanjiang | 59658 |
|  | Dongguan | 59289 |
|  | Jiangmen | 59478 |
|  | Shantou | 59316 |
|  | Shenzhou | 59493 |
|  | Zhongshan | 59485 |
|  | Zhuhai | 59488 |
| Anhui | Bengbu | 58221 |
|  | Bozhou | 58102 |
|  | Chizhou | 58419 |
|  | Chuzhou | 58236 |
|  | Fuyang | 58203 |
|  | Hefei | 58321 |
|  | Huainan | 58215 |
|  | Liuan | 58311 |
|  | Maanshan | 58336 |
|  | Suzhou | 58122 |
|  | Xuancheng | 58436 |
| Guangxi | Guilin | 57957 |
| Beijing | Beijing | 54511 |
| Chongqing | Chongqing | 57536 |
| Fujian | Putian | 58936 |
|  | Fuzhou | 58847 |
|  | Nanping | 58834 |
|  | Ningde | 58846 |
|  | Quanzhou | 59133 |
|  | Sanming | 58921 |
|  | Zhangzhou | 59126 |
| Gansu | Dingxi | 52996 |
| Guizhou | Zunyi | 57606 |
| Hebei | Baoding | 54602 |
|  | Chengde | 54423 |
|  | Shijiazhuang | 53698 |
|  | Handan | 53698 |
|  | Qinhuangdao | 54449 |
|  | Tangshan | 54534 |
|  | Zhangjiakou | 54401 |
| Heilongjiang | Daqing | 50950 |
|  | Haerbin | 50953 |
|  | Hegang | 50778 |
|  | Jiamusi | 50873 |
|  | Qiqihaer | 50745 |
|  | Jixi | 50978 |
|  | Mudanjiang | 54094 |
|  | Shuangyashan | 50888 |
|  | Yichun | 50774 |
| Henan | Anyang | 53898 |
|  | Pingdingshan | 57181 |
|  | Xuchang | 57089 |
|  | Kaifeng | 57091 |
|  | Luohe | 58111 |
|  | Nanyang | 57178 |
|  | Shangqiu | 58005 |
|  | Xinyang | 57297 |
|  | Xinxiang | 53986 |
|  | Zhengzhou | 57083 |
|  | Zhoukou | 57193 |
|  | Zhumadian | 57290 |
| Hubei | Huangshi | 58407 |
|  | Xianning | 57583 |
|  | Jingmen | 57378 |
|  | Jingzhou | 57476 |
|  | Wuhan | 57494 |
|  | Xiangyang | 57265 |
|  | Xiaogan | 57482 |
| Hunan | Changde | 57662 |
|  | Changsha | 57687 |
|  | Chenzhou | 57972 |
|  | Huaihua | 57749 |
|  | Hengyang | 57872 |
|  | Loudi | 57774 |
|  | Shaoyang | 57766 |
|  | Xiangtan | 57679 |
|  | Yiyang | 57671 |
|  | Yongzhou | 57866 |
|  | Yueyang | 57584 |
|  | Zhuzhou | 57780 |
| Jiangsu | Changzhou | 58343 |
|  | Lianyungang | 58040 |
|  | Nanjing | 58238 |
|  | Suqian | 58038 |
|  | Xuzhou | 58027 |
|  | Yancheng | 58150 |
|  | Zhenjiang | 58252 |
| Jiangxi | Fuzhou | 58715 |
|  | Jian | 57799 |
|  | Jiujiang | 58506 |
|  | Nanchang | 58606 |
|  | Shangrao | 58637 |
|  | Xinyu | 58608 |
|  | Yichun | 57793 |
|  | Yingtan | 58626 |
| Jilin | Baicheng | 50936 |
|  | Baishan | 54276 |
|  | Changchun | 54161 |
|  | Jilin | 54171 |
|  | Liaoyuan | 54260 |
|  | Siping | 54157 |
|  | Songyuan | 50948 |
|  | Tonghua | 54363 |
|  | Yanbian | 54186 |
| Liaoning | Anshan | 54339 |
|  | Benxi | 54346 |
|  | Chaoyang | 54563 |
|  | Shenyang | 54342 |
|  | Dalian | 54662 |
|  | Dandong | 54497 |
|  | Fushun | 54351 |
|  | Huhudao | 54452 |
|  | Jinzhou | 54337 |
|  | Panjin | 54470 |
|  | Tieling | 54254 |
|  | Yingkou | 54476 |
| Inner Mongolia | Hulun Buir | 50425 |
|  | Hinggan League | 50727 |
| Shandong | Binzhou | 54725 |
|  | Dongying | 54744 |
|  | Heze | 54909 |
|  | Jinan | 54823 |
|  | Qingdao | 54857 |
|  | Jining | 54916 |
|  | Linyi | 54929 |
|  | Rizhao | 54945 |
|  | Taian | 54826 |
|  | Weifang | 54843 |
|  | Weihai | 54774 |
|  | Yantai | 54751 |
|  | Zaozhuang | 54916 |
|  | Zibo | 54836 |
| Shaanxi | Baoji | 57025 |
|  | Xianyang | 57030 |
|  | Shangluo | 57143 |
|  | Tongchuan | 57037 |
|  | Weina | 57046 |
|  | Xian | 57131 |
|  | Yanan | 53845 |
|  | Yuncheng | 53959 |
| Sichuan | Liangshan Yi Autonomous Prefecture | 56479 |
| Tianjin | Tianjin | 54525 |
| Yunnan | Chuxiong Yi Autonomous Prefecture | 56763 |
| Zhejiang | Hangzhou | 58457 |
|  | Huzhou | 58450 |
|  | Jinhua | 58549 |
|  | Lishui | 58646 |
|  | Ningbo | 58467 |
|  | Quzhou | 58633 |
|  | Shaoxing | 58553 |
|  | Taizhou | 58652 |
|  | Wenzhou | 58752 |

Site number: the number of meteorological monitoring stations in China cities

**Table S2: The QAIC and QBIC of different lag months**

| Lag month | Mid temperature zone | | Warm temperate zone | | Subtropical zone | |
| --- | --- | --- | --- | --- | --- | --- |
|  | QAIC | QBIC | QAIC | QBIC | QAIC | QBIC |
| 1 | 13716.95 | 15405.7 | 36654.28 | 40415.77 | 30764.8 | 32624.41 |
| 2 | 13250.97 | 14884.37 | 34838.68 | 38345.05 | 30237.09 | 32064.63 |
| 3 | 12927.34 | 14527.48 | 32919.9 | 36116.52 | 29585.44 | 31377.32 |
| 4 | 12708.45 | 14298.8 | 31683.34 | 34732.56 | 29084.41 | 30848.54 |
| 5 | 12655.31 | 14277.58 | 30864.81 | 33827.84 | 28632.19 | 30389.12 |
| 6 | 12434.73 | 14037.96 | 29726.84 | 32564.82 | 28151.67 | 29891.58 |

QAIC: quasi-Akaike information criterion

QBIC: quasi-Bayesian information criterion

**Table S3: The lag effect between mean temperature, precipitation, relative humidity and HFRS infection**

| Lag | Temperature(P_2.5_) | Temperature(P_97.5_) | Precipitation(P_2.5_) | Precipitation(P_97.5_) | Relative humidity(P_2.5_) | Relative humidity(P_97.5_) |
| --- | --- | --- | --- | --- | --- | --- |
| 0 | 1.72 (1.31,2.25) | 1.26 (0.99,1.61) | 0.89 (0.78,1.01) | 0.90 (0.80,1.02) | 1.02 (0.89,1.16) | 0.88 (0.80,0.98) |
| 1 | 0.75 (0.60,0.93) | 0.40 (0.33,0.49) | 0.92 (0.83,1.03) | 1.05 (0.94,1.17) | 1.02 (0.91,1.13) | 0.79 (0.73,0.86) |
| 2 | 0.77 (0.68,0.86) | 0.61 (0.54,0.69) | 0.99 (0.92,1.07) | 1.00 (0.92,1.08) | 0.92 (0.85,1.00) | 0.93 (0.88,0.99) |
| 3 | 0.96 (0.86,1.08) | 1.16 (1.04,1.30) | 1.05 (0.97,1.13) | 0.94 (0.87,1.02) | 0.85 (0.79,0.93) | 1.09 (1.03,1.15) |
| 4 | 1.03 (0.91,1.17) | 1.34 (1.20,1.50) | 1.07 (0.99,1.15) | 0.95 (0.88,1.03) | 0.86 (0.79,0.93) | 1.11 (1.05,1.17) |
| 5 | 0.99 (0.91,1.08) | 1.10 (1.01,1.19) | 1.05 (0.99,1.12) | 1.03 (0.97,1.09) | 0.90 (0.84,0.97) | 1.03 (0.98,1.07) |
| 6 | 0.90 (0.73,1.11) | 0.76 (0.64,0.90) | 1.03 (0.92,1.15) | 1.14 (1.03,1.26) | 0.98 (0.87,1.10) | 0.91 (0.83,0.99) |

**Table S4**: **Model test of the interaction analysis between relative humidity and temperature in mid temperate zone**

| Variables | Edf | Ref.df | F-value | P-value |
| --- | --- | --- | --- | --- |
| Relative  humidity, Temperature | 23.080 | 26.976 | 16.289 | <0.001 |
| Month | 4.973 | 6.054 | 4.768 | <0.001 |
| Precipitation | 3.331 | 4.231 | 8.229 | <0.001 |

Abbreviations: Edf, effective degrees of freedom; Ref.df, reference degrees of freedom; F-value is the value of variables using F test.

**Table S5: Model test of the interaction analysis between precipitation and temperature in mid temperate zone**

| Variables | Edf | Ref.df | F-value | P-value |
| --- | --- | --- | --- | --- |
| Precipitation, Temperature | 23.364 | 27.028 | 11.974 | <0.001 |
| Month | 5.648 | 6.776 | 3.580 | <0.001 |
| Relative humidity | 5.620 | 6.809 | 7.198 | <0.001 |

Abbreviations: Edf, effective degrees of freedom; Ref.df, reference degrees of freedom; F-value is the value of variables using F test.

**Table S6: Model test of the interaction analysis between precipitation and relative humidity in mid temperate zone**

| Variables | Edf | Ref.df | F-value | P-value |
| --- | --- | --- | --- | --- |
| Precipitation, Relative humidity | 17.688 | 22.387 | 5.851 | <0.001 |
| Month | 5.655 | 6.780 | 3.690 | <0.001 |
| Temperature | 7.689 | 8.569 | 36.174 | <0.001 |

Abbreviations: Edf, effective degrees of freedom; Ref.df, reference degrees of freedom; F-value is the value of variables using F test.

**Table S7: Model test of the interaction analysis between relative humidity and temperature in warm temperate zone**

| Variables | Edf | Ref.df | F-value | P-value |
| --- | --- | --- | --- | --- |
| Relative  humidity, Temperature | 26.281 | 28.540 | 13.065 | <0.001 |
| Month | 8.899 | 8.996 | 33.426 | <0.001 |
| Precipitation | 5.957 | 7.074 | 7.552 | <0.001 |

Abbreviations: Edf, effective degrees of freedom; Ref.df, reference degrees of freedom; F-value is the value of variables using F test.

**Table S8: Model test of the interaction analysis between precipitation and temperature in warm temperate zone**

| Variables | Edf | Ref.df | F-value | P-value |
| --- | --- | --- | --- | --- |
| Precipitation, Temperature | 25.252 | 27.861 | 12.477 | <0.001 |
| Month | 8.933 | 8.998 | 38.377 | <0.001 |
| Relative humidity | 6.853 | 7.962 | 3.817 | <0.001 |

Abbreviations: Edf, effective degrees of freedom; Ref.df, reference degrees of freedom; F-value is the value of variables using F test.

**Table S9: Model test of the interaction analysis between precipitation and relative humidity in warm temperate zone**

| Variables | Edf | Ref.df | F-value | P-value |
| --- | --- | --- | --- | --- |
| Precipitation, Relative humidity | 24.849 | 27.686 | 7.039 | <0.001 |
| Month | 8.923 | 8.998 | 37.630 | <0.001 |
| Temperature | 7.748 | 8.606 | 31.439 | <0.001 |

Abbreviations: Edf, effective degrees of freedom; Ref.df, reference degrees of freedom; F-value is the value of variables using F test.

**Table S10: Model test of the interaction analysis between relative humidity and temperature in subtropical zone**

| Variables | Edf | Ref.df | F-value | P-value |
| --- | --- | --- | --- | --- |
| Relative  humidity, Temperature | 25.160 | 28.121 | 13.065 | <0.001 |
| Month | 7.559 | 8.471 | 6.802 | <0.001 |
| Precipitation | 5.634 | 6.827 | 1.422 | 0.187 |

Abbreviations: Edf, effective degrees of freedom; Ref.df, reference degrees of freedom; F-value is the value of variables using F test.

**Table S11: Model test of the interaction analysis between precipitation and temperature in subtropical zone**

| Variables | Edf | Ref.df | F-value | P-value |
| --- | --- | --- | --- | --- |
| Precipitation, Temperature | 17.791 | 22.335 | 4.097 | <0.001 |
| Month | 7.779 | 8.607 | 6.432 | <0.001 |
| Relative humidity | 8.123 | 8.794 | 5.444 | <0.001 |

Abbreviations: Edf, effective degrees of freedom; Ref.df, reference degrees of freedom; F-value is the value of variables using F test.

**Table S12: Model test of the interaction analysis between precipitation and relative humidity in subtropical zone**

| Variables | Edf | Ref.df | F-value | P-value |
| --- | --- | --- | --- | --- |
| Precipitation, Relative humidity | 24.598 | 27.750 | 1.813 | 0.006 |
| Month | 6.922 | 7.994 | 7.659 | <0.001 |
| Temperature | 7.696 | 8.577 | 14.684 | <0.001 |

Abbreviations: Edf, effective degrees of freedom; Ref.df, reference degrees of freedom; F-value is the value of variables using F test.
